# Quantification of Process Lethality (5-Log Reduction) of *Salmonella* and Salt Concentration during Sodium Replacement in Biltong Marinade

**DOI:** 10.3390/foods9111570

**Published:** 2020-10-29

**Authors:** Caitlin Karolenko, Peter Muriana

**Affiliations:** 1 Robert M. Kerr Food & Agricultural Products Center, Oklahoma State University, Stillwater, OK 74078, USA; caitlin.e.karolenko@okstate.edu; 2 Department of Animal and Food Sciences, Oklahoma State University, Stillwater, OK 74078, USA

**Keywords:** biltong, dried beef, salt replacement, *Salmonella*, 5-log reduction, marinade, potassium chloride, calcium chloride

## Abstract

Salt (sodium chloride, NaCl) is commonly used in ready-to-eat (RTE) meat products such as biltong, a South African style dried beef product for flavor, enhanced moisture loss, and reduction of microbial growth. However, increased consumption of high sodium content foods is commonly associated with high blood pressure and heart disease. This study evaluated the use of alternative salts, potassium chloride (KCl) and calcium chloride (CaCl_2_) in the biltong marinade to achieve a ≥ 5-log reduction of *Salmonella*, a pathogen of concern in beef products. Beef pieces (1.9 cm × 5.1 cm × 7.6 cm) were inoculated with a five-serovar mixture of *Salmonella* (*Salmonella* Thompson 120, *Salmonella* Enteritidis H3527, *Salmonella* Typhimurium H3380, *Salmonella* Heidelberg F5038BG1, and *Salmonella* Hadar MF60404), vacuum-tumbled in a traditional biltong marinade of salt, spices, and vinegar containing either NaCl, KCl or CaCl_2_ (2.2% concentration) followed by an 8–10 day drying period at 23.9 °C (75 °F) and 55% relative humidity. Microbial enumeration of *Salmonella* was conducted following inoculation, after marination, and after 2, 4, 6, 8, and 10 days of drying in a humidity/temperature chamber. Biltong produced with CaCl_2_, NaCl, or KCl achieved a > 5-log reduction of *Salmonella* after 6, 7, and 8 days, respectively. The *Salmonella* reduction trends with biltong made with NaCl or CaCl_2_ were not significantly different (*p* < 0.05) while both were significantly different from that made with KCl (*p* > 0.05). Sodium, calcium, and potassium ion concentrations were measured using ion-specific electrode meters following biltong processing and drying. As expected, the biltong made with the corresponding salt had the most abundant ion in the sample. Regardless of the salt used in the marinade, the potassium ion levels were moderately elevated in all samples. This was determined to be from potassium levels naturally present in beef rather than from other ingredients. Sampling of several commercial brands of biltong for sodium content showed that some were significantly above the allowable level of claims made on package ingredient statements. The substitution of NaCl with KCl or CaCl_2_ during biltong processing can also provide a 5-log reduction of *Salmonella* to produce a safe product that can be marketed as a more healthy low-sodium food alternative that may appeal to consumers who need to reduce their blood pressure and are conscientious of sodium levels in their diet.

## 1. Introduction

Salt has historically been used for hundreds of years in food for two main purposes: flavoring and preservation. However, sodium intake among consumers has dramatically increased with the consumption of processed foods [1]. High sodium intake is associated with many health issues including hypertension and cardiovascular disease [2,3,4]. The recommended dietary guidelines for intake of sodium should be less than 2300 milligrams (mg), however the average adult normally consumes more than 3000 mg per day [5]. The World Health Organization and the American Heart Association recommend even lower levels, of less than 2000 and 1500 mg/day, respectively. Therefore, it is important for consumers to have options for food products that contain low levels of salt without compromising on the taste or texture of the food. Aside from the health consequences of an overabundance of sodium in a typical US diet, potassium and calcium deficiencies are a growing health concern for consumers. Various food items attempt to supplement these nutrients with claims of being ‘fortified’. The replacement of NaCl during the production of biltong with other salts such as KCl or CaCl_2_ could serve as an alternative source for these nutritive ions which are lacking in many consumers’ diet.

High sodium content is of particular importance for processed, dried or cured meats that traditionally rely on the addition of sodium chloride (NaCl) during processing to help flavor and preserve the meat. Salt contributes to the decrease in water activity (a_w_) by drawing water out of the meat, limiting the amount of free water available for microbial growth as well as influencing the overall flavor, appearance, and texture of the product [6]. The addition of salts to dried meats can aid in interference with bacterial cellular mechanisms thus reducing the rate of bacterial growth [7]. One strategy to reduce the sodium content in foods while preserving the microbiological inhibitory effects is to replace NaCl with an alternative salt such as KCl, CaCl_2_, or MgCl_2_ [8,9]. Replacing sodium with alternative salts can also potentially help to achieve a healthier food product while still maintaining the same quality and safety. The concept of salt replacement in processed meat products is not new, as many studies have examined the replacement of NaCl with KCl, CaCl_2_, or MgCl_2_ in dried meat products [10,11,12]. Partial replacement of NaCl with KCl and CaCl_2_ in various dried meat products including dry-cured pork loins and Spanish dry-cured hams demonstrated similar organisms as traditional products made with NaCl and no significant differences in a_w_ [10,11,12]. Although sensory attributes of salt and salt replacement are important in all meat products, they are more readily detected in large, intact muscle products (dry-cured hams) where the final product is associated with a recognized and expected meat flavor and where salt type can influence lipid oxidation over the extended drying period [13,14]. However, smaller and thin-shaved meat products (jerky, biltong) all have numerous product variations and a high degree of topical seasoning that may mask alternative salt sensory attributes. From a microbial safety standpoint, these studies have shown that it is possible to achieve a microbially safe, dry cured product with a partial or total alternative salt replacement similar to those that are produced with NaCl.

Biltong, is a South African style dried beef (jerky, kippered beef) product that uses lean cuts of beef that are marinaded in a traditional spice and vinegar mixture which includes salt and is then dried for an extended period of time at ambient temperature [15,16,17]. In the United States (US), the US Department of Agriculture Food Safety and Inspection Service (USDA-FSIS) requires manufacturers of biltong to provide microbial validation of reduction of *Salmonella* (a foodborne pathogen often associated with raw meat products) by one of two approaches [18]. One approach (“process reduction + *Salmonella* testing”) is to demonstrate ≥ 2-log reduction of *Salmonella* by the process while simultaneously testing each lot of ingredients for *Salmonella* to insure they are *Salmonella*-free; the other method (“process-only”) is to ensure that the process itself (without *Salmonella*-testing of ingredients) demonstrates a ≥ 5-log reduction of *Salmonella* [18]. This latter process is the most sought after because continuous testing of ingredients for *Salmonella* in the former process is laborious and expensive, and if ingredients test positive, they cannot be used unless rendered free of *Salmonella*.

Currently, there is no known published research available that identifies whether the use of alternative salts in biltong manufacture has the same ability to contribute to the reduction of foodborne pathogens during biltong processing as does traditional salt (NaCl). In this study, we examined the efficacy of using alternative salts including KCl and CaCl_2_ in place of traditionally used NaCl during biltong processing to achieve a USDA-FSIS required ≥ 5-log reduction of *Salmonella* if using the “process-only” method of biltong manufacture.

## 2. Materials and Methods

### 2.1. Bacterial Strains and Growth and Storage Conditions

Acid-adapted *Salmonella* serovars used in this study included: *Salmonella enterica* subsp. *enterica* serotype Thompson 120 (chicken isolate), *Salmonella enterica* subsp. *enterica* serotype Hadar MF60404 (turkey isolate), *Salmonella enterica* subsp. *enterica* serotype Heidelberg F5038BG1 (ham isolate), *Salmonella enterica* subsp. *enterica* serotype Typhimurium H3380 (DT 104 clinical isolate), and *Salmonella enterica* subsp. *enterica* serotype Enteritidis H3527 (phage type 13a, clinical isolate). These strains have been described in numerous research publications involving antimicrobial processing interventions against *Salmonella* spp [19,20,21,22,23,24].

*Salmonella* cultures were inoculated into Tryptic Soy Broth (TSB, BD Bacto, Franklin Lakes, NJ, USA) and grown at 37 °C. Cultures were prepared for storage by centrifugation (6000× *g*, 5 °C) of 9–10 mL of fresh, overnight cultures and resuspending the resulting cell pellets with 2–3 mL of fresh sterile TSB containing 10% glycerol. The resuspended cells/freezing menstrum were placed in glass vials and stored in an ultra-low freezer (−80 °C). Frozen stocks were revived by transfer of 100 µL of thawed cell suspension into 9 mL of TSB, incubating overnight at 37 °C, and sub-culturing twice before use. Serial dilutions were made in 0.1% Buffered Peptone Water (BPW, BD Difco) and microbial enumeration was carried out on Tryptic Soy Agar (TSA, BD Bacto; 1.5% agar), plated in duplicate.

Acid adaptation of these *Salmonella* serovars was carried out with cultures inoculated into TSB augmented with 1% glucose according to Wilde et al. and others [23,24,25,26]. Individual cultures were harvested by centrifugation, resuspended with 0.1% BPW (BD Difco), and held at refrigerated temperature until use (5 °C). The centrifuged and resuspended individual cultures were then combined in equal proportions to obtain the mixed inoculum.

### 2.2. Beef Processing and Inoculation

Intact, select grade, beef bottom-round sub-primal cuts (Ralph’s Packing Co., Perkins, OK, USA) were trimmed to approximately 5.1 cm wide × 1.9 cm thick × 7.6 cm long beef pieces at the R.M. Kerr Food and Agricultural Product Center (FAPC). Beef pieces used for this experiment were vacuum-packaged fresh, flash frozen (−80 °C), stored frozen (−20 °C), and thawed immediately before use. The beef pieces were inoculated by pipette with 150 uL of the 5-serovar *Salmonella* inoculum mixture on each side, and immediately spread with a ‘gloved finger’. Inoculated beef pieces were then incubated for 30 min at 4–5 °C to allow for bacterial attachment prior to use.

### 2.3. Biltong Processing and Salt Replacement

Inoculated beef pieces were placed in plastic dip cages and dipped in sterilized water for 30 s (to mimic wetting by rinse treatment and/or alternative antimicrobial dips) and excess liquid was allowed to drain off. Meat pieces were then transferred to chilled steel tumbling vessels containing a biltong marinade. The biltong marinade was comprised of 2.2% salt, 0.8% black pepper, 1.1% coarse ground coriander and 4% red wine vinegar (100-grain; 10% acetic acid) as a percentage of total meat weight. Separate marinades were formulated to determine the efficacy of alternative salts in the biltong process whereby 2.2% NaCl (Fisher Chemical, Fisher Scientific, Atlanta, GA, USA) was replaced with 2.2% KCl (Fisher Chemical), or 2.2% CaCl_2_ (Acros Organics, Fisher Scientific). Beef pieces were sealed in a steel drum with marinade, a vacuum was drawn to 15 inches Hg and then locked, and the drum was allowed to rotate for 30 min on a vacuum tumbler (Biro VTS-43, Marblehead, OH, USA). Meat pieces were then hung in a temperature-controlled humidity oven (Hotpack, Warminster, PA, USA) maintained at 55% relative humidity (RH) and 23.9 °C dried for up to 10 days. Sampling was conducted following marination (Day 0) and then again every 48 h.

For comparative purposes, the pH and a_w_ of raw beef starting product and 8-day product were compared. Analysis of a_w_ was performed as previously described and included both internal a_w_ of sliced biltong beef pieces as well as ground pieces [24]. The pH of raw beef was performed directly after grinding the raw beef, or with dried beef by adding 2 parts deionized water to 1 part dried beef (ground) for samples that had been dried for 8 days.

### 2.4. Microbial Anaylsis

At each sampling time point, meat pieces were randomly selected from each salt marination batch and transferred to sterile Whirl-pak filter-stomaching bags (4-mil; Nasco, Fort Atkinson, WI, USA). After addition of 100 mL of 1% neutralizing Buffered Peptone Water (nBPW, Criterion, Hardy Diagnostics, Santa Maria, CA, USA), samples were stomached for 90 s in a Masticator paddle-blender (IUL Instruments, Barcelona, Spain). Serial dilutions were surface plated on Selenite Cystine Agar (SCA) containing spectinomycin (5 μg/mL), clindamycin (5 μg/mL) and novobiocin (50 μg/mL) as described previously by Karolenko et al. [23]. The filter bag dilution was considered the 10^0^ dilution. Plates were incubated for 48 h at 37 °C and enumerated as log CFU/mL relative to the 10^0^ filter bag dilution. Treatments were performed in duplicate replication and sampled in triplicate at each sampling time.

### 2.5. Determination of Salt Ion Concentration in Experimental Biltong and Comparison to That in Commercially Available Biltong

**Figure 1:**
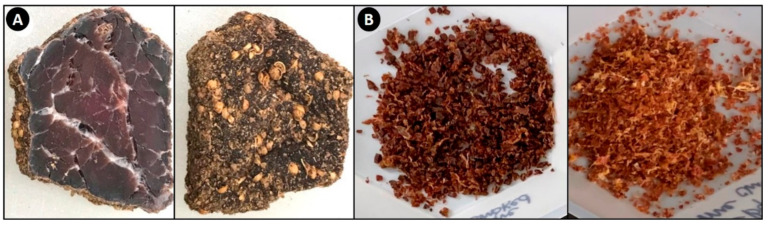
Sampling biltong for salt ion analyses: (**A**) representative in-house manufactured biltong after 8 days of drying showing the inside of dried beef muscle tissue (left) and retention of surface seasoning (right); (**B**) finely ground biltong samples to be mixed in water for ion analysis.

Following drying, biltong pieces (Figure 1A) were cut into smaller pieces and finely ground using a laboratory blender (Waring Commerical, New Harford, CT) until a homogenized mixture was formed (Figure 1B). Five (5) g of the finely ground dried beef was weighed out and brought up to 100 g with distilled water in a stomacher bag and macerated in a paddle mixer to homogenize the sample thoroughly.

Individual Horiba LAQUA Twin Pocket Ion Meters (Horiba Intruments, Irvine, CA, USA) were used for Na^+^, Ca^2+^, and K^+^ ion quantitation. Although the instruments came with an ion standard solution, we also prepared a series of standards that would reflect the various levels experienced in our testing to ensure the linearity of the response throughout this range. As per the manufacturer’s instructions, 300 μL of the diluted/mixed sample was placed into the sensor chamber of the appropriate salt meter and stable readings (in ppm) were recorded. Readings were taken in duplicate from three different batch trials. Additionally, each sample was also tested in the remaining two ion meters to confirm the presence or lack of any additional salt ions. To determine the ion concentration content per serving size, the following equation was used:

(1) $$\mathrm{Calculated}\;\mathrm{Ion}\;\mathrm{ppm}\;\left(\mathrm{mg}/100\;g\right)=\mathrm{Meter}\;\mathrm{reading}\;\left(\frac{\mathrm{mg}}{L}\right)\times \frac{\mathrm{Volume}\;\mathrm{after}\;\mathrm{Dilution}\;\left(L\right)}{\mathrm{Biltong}\;\mathrm{Weight}\;\left(g\right)}\times 100$$

(2) $$\mathrm{Calculated}\;\mathrm{Ion}\;\mathrm{per}\;\mathrm{Serving}\;\mathrm{Size}\;\left(\frac{\mathrm{mg}}{28\;g}\right)=\mathrm{Calculated}\;\mathrm{Ion}\;\left(\frac{\mathrm{mg}}{100\;g}\right)/3.57\;$$

The serving size was determined by using the commonly-used serving sizes listed on commercial biltong products that list a serving size of 28 g (1 oz).

Ion concentrations (Na^+^, Ca^2+^, K^+^) of commercially produced biltong were sampled using the protocol listed in Section 2.5 with slight modifications. Two different formulations of biltong products were tested from each of two different biltong manufacturers (i.e., Company A, Company B) for a total of four products analyzed in total. Samples were ground until a fine uniform consistency was achieved using the laboratory blender (Figure 1B). Samples were processed as described above and ion concentrations measured with each of the ion meters. Three different ground mixtures were made from each type of biltong product and then readings were averaged. Readings for each sample taken with the ion meters were made from duplicate samplings. Percent recovery of sodium was calculated by:

(3) $$\mathrm{Percent}\;\mathrm{Recovery}\;\left(\mathrm{Sodium}\right)=\frac{\mathrm{Calculated}\;\mathrm{Na}\;\left(\frac{\mathrm{mg}}{28\;g}\right)}{\mathrm{Listed}\;\mathrm{Sodium}\;\mathrm{on}\;\mathrm{Label}\;\left(\frac{\mathrm{mg}}{28\;g}\right)}$$

Calcium and potassium ion concentrations of each biltong product were also determined using the equations in Section 2.5.

### 2.6. Statistical Anaylsis

Replicate process validation trials were performed in duplicate, with 3 samples tested per sampling period within each replication (*n* = 6), in accordance with inoculated validation criteria established by the National Advisory Committee on Microbial Criteria for Foods (NACMCF) [27] and accepted by USDA-FSIS [28]. Replications were performed as autonomous and separate experiments using separately-inoculated cultures and prepared plating media. All other tests were performed in triplicate replication. All data are presented as the mean of multiple replications with standard deviation of the mean represented by error bars. Statistical analysis of timed series data was performed using repeated measures one-way analysis of variance (RM-ANOVA); statistical analysis of all other data was done using one-way analysis of variance (ANOVA). Pairwise multiple comparisons were done using the Holm–Sidak test to determine significant differences. Data treatments with the same letter are not significantly different (*p* > 0.05); treatments with different letters are significantly different (*p* < 0.05).

## 3. Results

### 3.1. Microbial Lethality Validation of Salt Replacement

**Figure 2:**
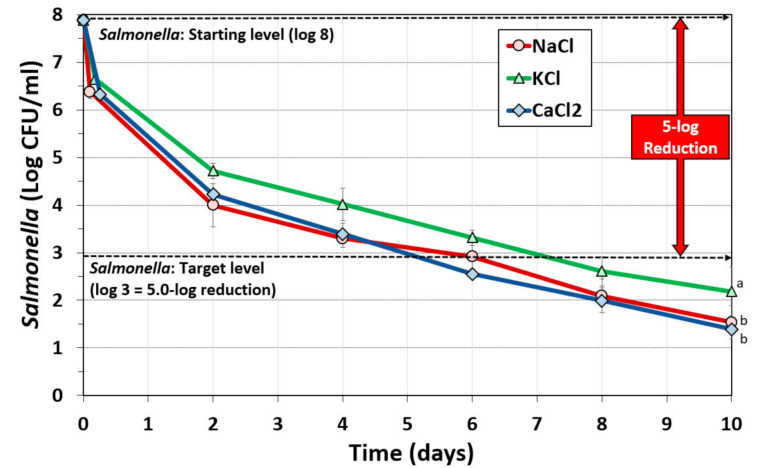
Biltong processing using alternative salts for reduction of *Salmonella.* Comparison of NaCl, KCl, and CaCl_2_ at a concentration of 2.2% to attempt a 5-log reduction of *Salmonella* population over a period of ten days at 23.9 °C (75 °F) and 55% relative humidity (RH). Statistical analysis of entire time course of graphs: treatments with the same letter are not significantly different (*p* > 0.05); treatments with different letters are significantly different (*p* < 0.05).

A 2.2% salt concentration was applied in the ingredient formulation for all three salts (NaCl, KCl, or CaCl_2_) used in separate biltong marinades for comparison of their effect on reduction of *Salmonella* during biltong processing. All inoculated beef pieces were subjected to a 30 s rinse treatment in sterile water to mimic commercial rinse practices, resulting in ~0.2-log reduction of the inoculated *Salmonella* (not shown on graph; combined with post-marinade reduction). Subsequent marination of beef pieces with NaCl had a post-marinade reduction of 1.38-log while treatments using KCl and CaCl_2_ had a post-marinade reduction of 1.11- and 1.43-log, respectively (Figure 2). During the drying of biltong over the next 4 days, *Salmonella* levels continued to decline at a similar rate (Figure 2). Biltong formulated with CaCl_2_ was able to achieve ≥ 5-log reduction by day 6, biltong formulated with NaCl achieved this same benchmark by day 7 (by extrapolation), and that made with KCl by day 8. By the end of 10 days of drying, biltong made with either CaCl_2_ or NaCl achieved an overall reduction of *Salmonella* of 6.37-log and 6.22-log, respectively, while that made with KCl achieved a 5.57-log reduction over the same time period. Statistical analysis of these three biltong processes demonstrated that the NaCl and CaCl_2_ formulations were not significantly different from each other (*p* > 0.05), but they were significantly different than biltong formulated with KCl (*p* < 0.05). In spite of these differences, all three formulations achieved > 5-log reduction of *Salmonella* within 8-days.

### 3.2. Determindation of Final Salt Ion Concentration, pH, and A_w_ in Biltong

**Figure 3:**
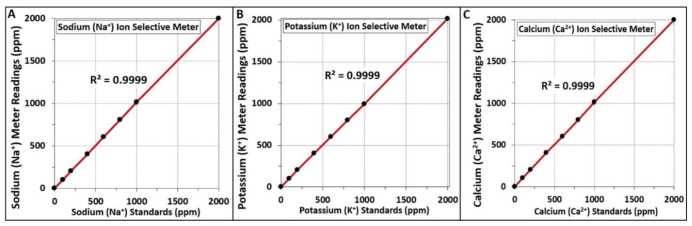
Ion analyses on standardized salt solutions using ion selective electrode (ISE) meters used in this study. Panel (**A**), ion analysis of Na^+^ using NaCl standards; (**B**), ion analysis of K^+^ using KCl standards; (**C**), ion analysis of Ca^2+^ using CaCl_2_ standards. Individual standards were prepared in triplicate and data points represent the mean of triplicate samplings with error bars representing the standard deviation of the mean. The coefficient of determination is a measure of the linearity of the data points to the trendline is indicated by R^2^ value.

Specific ion selective electrode (ISE) meters were used for analysis of Na^+^, Ca^2+^, and K^+^ ion levels. The testing of standardized salt solutions throughout the range that might be tested in our biltong samples gave excellent results (R^2^ values of 0.9999) for each range of standards used for the various individual ISE meters (Figure 3).

**Figure 4:**
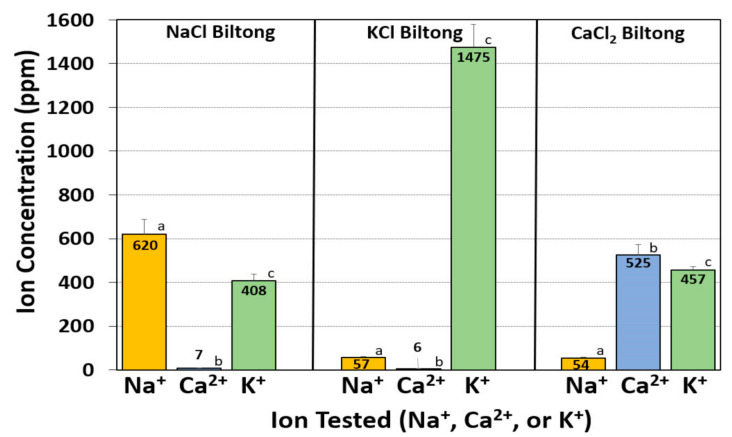
Analysis of Na^+^, Ca^2+^ and K^+^ ions in biltong made with NaCl, KCl, or CaCl_2_. Comparison of all 3 ion concentrations in in each batch of biltong made with a single added salt. Data are presented as the mean of triplicate replications and error bars represent the standard deviation from the mean. Statistical analysis was only performed on ion analyses within the same biltong salt formulation. Means with the same letter are not significantly different (*p* > 0.05) whereas means with different letters are significantly different (*p* < 0.05).

The ion corresponding to the appropriate salt that the marinade was formulated with was the most abundant ion in the sample (Figure 4). Biltong made with NaCl resulted in a Na^+^ concentration of 620 ppm, followed by K^+^ (408 ppm) and Ca^2+^ (7 ppm). Biltong made with KCl resulted in a K^+^ concentration of 1475 ppm followed by Na^+^ (57 ppm) and Ca^2+^ (5.8 ppm). Similarly, biltong made with CaCl_2_ resulted in a Ca^2+^ concentration of 525 ppm followed by K^+^ (457 ppm) and Na^+^ (54 ppm) (Figure 4).

**Figure 5:**
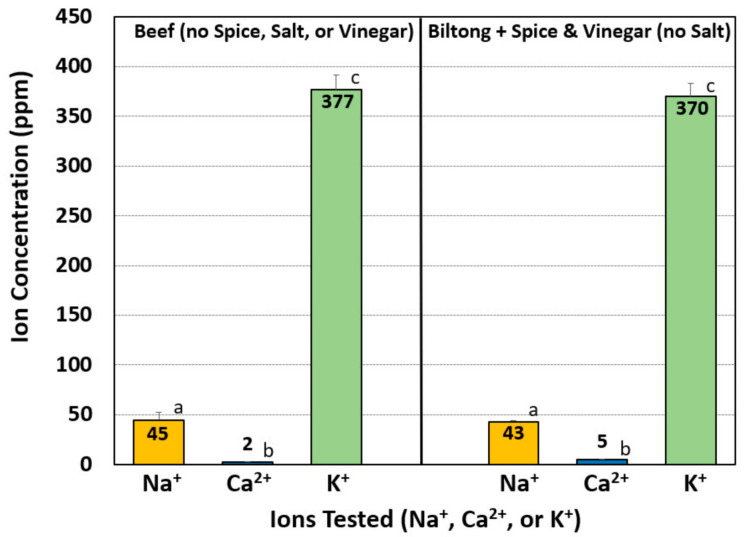
Comparison of ion concentrations of biltong made with dried beef without spice, salt, or vinegar and biltong made with only spice and vinegar marinade (without salt). Data are presented as the mean of triplicate replications, and error bars represent the standard deviation from the mean. Comparisons of means with different letters are significantly different (*p* < 0.05) as determined by one-way ANOVA using the Holm–Sidak test for pairwise multiple comparisons; means with the same letter are not significantly different (*p* > 0.05).

In order to determine the source of additional potassium, dried biltong beef was produced using unseasoned meat pieces without spices, vinegar, or salt, as well as meat pieces seasoned only with spices and vinegar (without salt). Both trials were then dried for 8 days, finely ground, and sampled for ion analysis as described previously. The resulting ion levels for both the unseasoned meat pieces as well as the spice/vinegar biltong pieces were low in both Na^+^ (43–45 ppm) and Ca^2+^ (1–5 ppm) ions, but high in K^+^ ions at 377 ppm and 370 ppm, respectively (Figure 5).

During biltong manufacture, several processing parameters converge to inhibit the *Salmonella* inoculum: acidic antimicrobials (antimicrobials/vinegar), salt, a_w_, and dessication. To further assess the contribution of acidic solution vs. drying on reduction of *Salmonella*, we examined pH and a_w_ at the beginning and end of the process. The pH of the initial raw beef was pH 5.5 and after marinade processing followed by 8-days of drying, the beef was still pH 5.0 (CaCl_2_ biltong), 5.26 (NaCl biltong), and 5.38 (KCl biltong) (Table 1). Similarly, the a_w_ of the initial raw beef was 0.9865 and after 8 days of processing/drying, the internal a_w_ of sliced biltong pieces was reduced to 0.8206 (NaCl), 0.8276 (CaCl_2_), and 0.8380 (KCl) (Table 1). The a_w_ of finely ground biltong beef pieces was significantly lower: 0.6690 (KCl), 0.6860 (CaCls), and 0.6879 (NaCl) (Table 1).

### 3.3. Salt Ion Analysis from Commercial (Retail) Biltong

**Figure 6:**
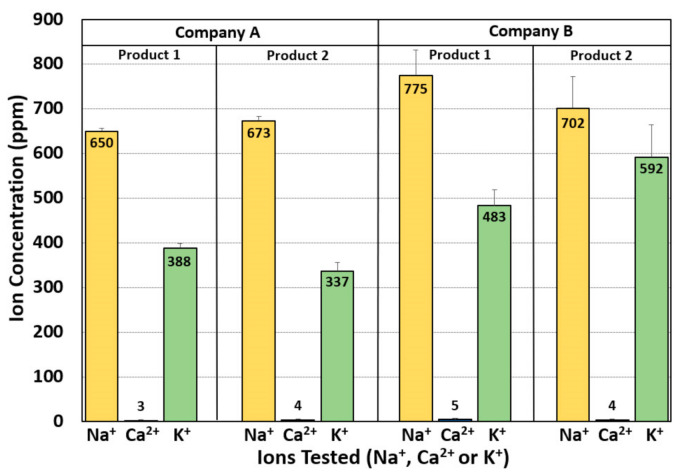
Salt ion analyses of two commercially-available biltong products (Product 1; Product 2) from each of two different companies (Company A; Company B). Analyses were done in triplicate on each product with errors bars indicating the standard deviation of the mean.

Samples of commercially available biltong from retail supermarkets were analyzed for various salt ion contents (Na^+^, Ca^2+^, K^+^; Figure 6) to compare with levels determined from our in-lab manufactured biltong (Figure 5). As with our own biltong made without CaCl_2_, all of the retail biltong samples had very low levels of Ca^2+^ (Figure 6). Additionally, similar elevated levels of K^+^ were observed in the commercial biltong (336–591 ppm) and compared favorably to our laboratory biltong which ranged from 370–456 ppm K^+^ (excluding the levels of K^+^ observed from biltong produced with KCl). The biltong made in this study using NaCl (Figure 4) demonstrated comparable Na^+^ concentrations (620 ppm) when compared to that produced by Company A (650, 673 ppm Na^+^), but had significantly lower levels than produced by Company B (702, 775 ppm Na^+^) (Figure 6).

**Figure 7:**
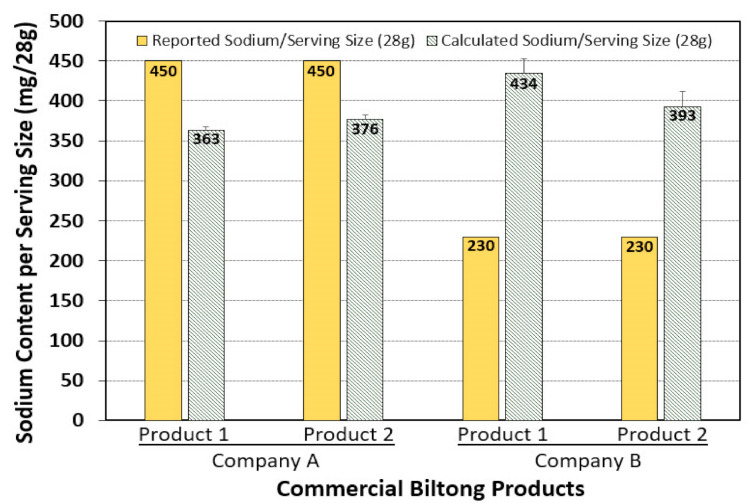
Comparison of reported Na^+^ content (per serving size) to the calculated Na^+^ content of two commercial biltong products (Product 1, 2) for each of two companies (Company A, B). Reported values were obtained from product labels; calculated values were based on triplicate replication of Na^+^ analyses using the method described herein. Products were obtained locally from supermarkets.

Additionally, the Na^+^ content per serving size (28 g) of each of the commercial biltong samples was calculated using the experimental Equation (3) and was compared to the reported sodium content on the dietary label. Both products from Company A over-stated the Na^+^ content on their label compared to the calculated (lower) Na^+^ content that was determined from their product using the ISE meters (Figure 7). Conversely, the products made by Company B significantly under-stated the Na^+^ content on their labels compared to the significantly higher Na^+^ content calculated by analyses of their products (Figure 6). Ion analyses done for experimental biltong produced in this study obtained similar values as Company A (i.e., 374 mg Na^+^/28 g biltong). Substitution of different salts for NaCl demonstrated significantly lower levels of Na^+^ per serving size of biltong: i.e., 30 mg Na^+^/serving when CaCl_2_ was used, or 32 mg Na^+^/serving when KCl was used (Table 2) while still achieving ≥ 5-log reduction of *Salmonella* (Figure 2).

## 4. Discussion

Biltong is a ready-to-eat (RTE) dried beef product that is not processed to as high a temperature as beef jerky. Rather, it is dried at moderate temperatures (i.e., 23.9 °C/75 °F) and relative humidity (~55% RH) and relies on salt, spices, and vinegar, along with extended drying (4–10 days) relative to jerky, to achieve USDA-FSIS recommended ≥ 5-log reduction of *Salmonella*. The use of acid-adapted *Salmonella* cultures ensures that the *Salmonella* inoculum is not easily inhibited by acid treatment with vinegar and/or other antimicrobials that are allowed during biltong processing [23]. The vinegar/acid treatment provided by a short term antimicrobial dip treatment (30–60 s) or meat marinade (i.e., 30 min) is effective as a microbial inhibitor during the short time the surface pH is lowered by vinegar or acid treatment. During the vinegar/marinade treatment, we observed approximately 1.1–1.43-log reduction of *Salmonella* that when combined with a pre-marinade rinse treatment (~0.2-log reduction), results in a post-marinade total reduction of ~1.31–1.63 log reduction (Figure 2). Upon removal from the marinade, residual moisture is absorbed, the vinegar is diluted by diffusion into the beef, and the acidic pH is buffered by the underlying mass of beef protein which equilibrates back to the normal pH of beef. Acid inhibition of surface bacteria is highest when there is sufficient acid to render a low surface pH level. Weak organic acids are more effective when the pH is below the pK_a_ of the acid (acetic acid pK_a_ = 4.76) at which they are more capable of diffusing into bacterial cells where they dissociate into the toxic anion adduct of the weak acid at the neutral pH of the bacterial cytoplasm that is well above the pK_a_ of the acid [29,30]. The acidic effect of marinade may be prolonged for those processes that have an extended/overnight marination [24]. In the current study, the marination was performed for 30 min and the beef was removed and hung to dry for up to 10 days at 23.9 °C (75 °F) and 55% RH. Biltong beef was examined for pH after 8-days of drying (to be consistent with our prior studies that stopped at 8 days) and, regardless of the salt used in the marinade, the pH was observed to be pH 5.00–5.30 (Table 1). This pH range is close to the isoelectric point of meat protein (pH 5.1–5.2) at which it has net zero charge and less water binding capacity than beef of higher or lower pH [31,32].

External salt on the beef provided by the marinade, and that which is absorbed into the beef periphery via vacuum tumbling, theoretically binds available water, and initiates the lowering of a_w_ as observed in prior post-marination a_w_ analyses [24]. This, together with drying conditions (temperature, RH, time), are present for the entire drying time during which biltong is usually harvested (6–10 days), and work in concert to lower the a_w_ of the product to limit growth and inhibit microorganisms [24]. Under these drying conditions, biltong results in ~60% moisture loss [24] and the relative concentration of salt likely increases to higher levels. In our study, the a_w_ of raw beef (0.9985) was reduced to 0.8206–0.8380 (internal) or 0.6690–0.6879 (ground) after 8 days of drying. Chopped samples are often used to obtain average a_w_ values. However, the internal a_w_ is a much more important parameter when vacuum tumbling is involved because of the potential to draw bacteria internally during this process [33,34,35]. Therefore, the USDA-FSIS considers vacuum tumbled beef as ‘non-intact’ beef product (similar to ground beef and blade/needle-tenderized beef) and the internal a_w_ must be <0.85 to prevent growth and enterotoxin production by *Staphylococcus aureus* should it be internalized during vacuum tumbling. In combination, these factors help to achieve the necessary ≥5-log reduction of *Salmonella* that is sought for a USDA-FSIS validated dried beef process [24].

Given the concerns for high sodium in our diets, questions have developed on whether alternative salts such as KCl or CaCl_2_ can provide an equivalent 5-log reduction of *Salmonella* while reducing the amount of sodium in the product. Using acid-adapted cultures to reduce the acid sensitivity of *Salmonella* [23], a >5-log reduction of *Salmonella* was obtained in 7-days using 2.2% NaCl and 4% vinegar as observed in this paper (Figure 2) and as described previously [24]. By replacing NaCl with either CaCl_2_ or KCl in the marinade formulation, we still achieved a >5-log reduction of *Salmonella* in 6 and 8 days, respectively (Figure 2). Although beef pieces were cut to similar sizes by hand, we have not determined whether slight differences in size could influence the rate of drying and affect the timeline of log reduction.

Salt ion concentrations (Na^+^, Ca^2+^ and K^+^) of biltong made with different salts were determined using ion selective electrode (ISE) meters. These meters have steadily improved during the last 30 years and compare favorably with more sophisticated instruments [36,37,38]. Although the meters provide direct readings as ‘ppm’, we further examined the accuracy of readings using standardized solutions throughout the entire range examined in this work. The meters demonstrated excellent accuracy and repeatability with R^2^ values of ≥0.9999 (Figure 3). As expected, the ion present in the highest concentration was the same ion from the salt added to the marinade. Surprisingly, K^+^ was not only high in biltong made with KCl, but it was also moderately high in biltong produced with the other salts (Figure 3). The unexpectedly high values of K^+^ present in all of the biltong samples, even when KCl was not added to the marinade, caused us to search for additional sources of K^+^ in the process. Testing of beef marinaded with just spices and vinegar (no salt), or just the dried beef alone, helped determine that the beef itself was a dominate source of K^+^ (Figure 4). There are numerous studies documenting moderately high levels of K^+^ within beef similar to the levels observed in the present study [39].

Salt ion levels were also examined for two flavored formulations produced by each of two manufacturers available at local supermarkets. We noticed high Na^+^ levels which was expected as “salt” is listed on the ingredient label and negligible Ca^2+^ levels. The retail brands were moderately high in K^+^ levels and is likely contributed by the beef source (Figure 5) as per our results with unseasoned dried beef (Figure 4). The ion levels in the retail brands were further re-calculated based on serving size (Figure 6) and then compared to our own biltong (Table 2). On a “per serving” basis (28 g, 1 oz), the products produced by Company A demonstrated lower Na+ levels (360 and 373 mg/serving) that resulted in 80% and 83% of the on-package ingredient label claims (450 mg/serving), respectively. Analyses of biltong made in our study (2.2% NaCl, 4% vinegar, spices) demonstrated levels comparable with Company A (374 mg/serving) (Table 2). Conversely, the products produced by Company B were 67% and 85% higher (393 and 434 mg/serving) than their ingredient labels claims for sodium (235 mg/serving) (Figure 6). The level of salt ion on the final product could readily be affected by a change in processing conditions such as injected marinade vs. dip marinade, fine salt vs. coarse granulated salt, vacuum-tumbling vs. no vacuum-tumbling, and short time marination vs. overnight margination, such that the final product no longer lives up to claims listed on the ingredient label. This can more readily occur when another company (i.e., a co-packer) manufactures for the seller. US Food and Drug Administration (FDA) regulations suggest that listed ingredients that occur at more than 20% less than, or more than 20% greater than, declared level claims (beyond the limits of analytical variability) the product could be deemed ‘misbranded’ [40].

Additional studies could examine the effects these salts have on the sensory characteristics of biltong including taste, texture and tenderness. Studies of salt substitution in other types of beef have described off-tastes such as “bitter” and “metallic” and an increase in hardness associated with CaCl_2_ and KCl at high concentrations [11,41]. However, some studies have indicated a preference of KCl over other alternative salts and/or no issues when limited to partial replacement of traditional NaCl [14,42] or masked when flavorings are added in marinades [41]. Although some of these meat products are significantly different than biltong, this may not be an issue given the strong spice profile of surface seasonings from the marinade on these types of products. As we strive to examine additional processing modifications that may also affect flavor, future studies could evaluate that the product is not only microbially safe for consumers but also tastes acceptable as there is not much work done in this area with biltong.

## 5. Conclusions

The replacement of NaCl with other ingredients such as KCl or CaCl_2_ in the biltong marinade resulted in a ≥ 5-log reduction of *Salmonella* to achieve the USDA-FSIS validation of biltong with either NaCl, CaCl_2_, or KCl, providing manufacturers a choice of alternative salt to reduce sodium content if desired. Although the objective of our work was to demonstrate that KCl or CaCl_2_ could also obtain a sufficient reduction of *Salmonella* to meet USDA-FSIS biltong validation criteria, there are also human health benefits to use of these different salts. Such alternative salts allow biltong to be produced and marketed as a more healthy low-sodium food alternative while simultaneously being considered to be “fortified” with either K^+^ or Ca^2+^ as consumers seek to supplement deficiencies of these minerals in their diet.

## Figures and Tables

**Table 1:** Initial and final pH and water activity (a_w_) of biltong beef (before processing and after 8 days of drying) made with NaCl, KCl, or CaCl_2_. All analyses were performed on a triplicate series of samples.

	pH of Biltong		A_w_ of Biltong		
Type of Biltong	Initial pH (Raw Beef)	Final pH (8 Days)	A_w_ Initial (Raw Beef)	A_w_ Final (8 Days) Internal	A_w_ Final (8 Days) Ground
Made using NaCl	5.50 ± 0.04	5.26 ± 0.06	0.9865 ± 0.0054	0.8206 ± 0.0150	0.6879 ± 0.0196
Made using CaCl_2_		5.38 ± 0.01		0.8380 ± 0.0032	0.6690 ± 0.0334
Made using KCl		5.00 ± 0.01		0.8276 ± 0.0141	0.6860 ± 0.0230

**Table 2:** Salt ion concentration of biltong beef produced during this study based on a comparable 28 g (1 oz) serving size.

	Avg/Serving (mg/28 g Biltong)		
Type of Biltong	Na^+^	Ca^2+^	K^+^
Made using NaCl	374.3	3.9	228.3
Made using CaCl_2_	30.3	293.5	255.3
Made using KCl	31.9	3.3	823.0
Made with unseasoned beef	25.0	0.4	211.0
Beef + spice, vinegar (no salt added)	23.9	2.5	206.9
